# Carbuncle: Its Pathology and Treatment

**Published:** 1868-01

**Authors:** John M. Johnson

**Affiliations:** Atlanta, Ga.


ATLANTA

Medical and Surgical Journal.

NEW SERIES.

Vol. VIII. JANUARY, 1868. No. 11.

### ORIGINAL COMMUNICATIONS.

### ARTICLE I.

*Carbuncle: Its Pathology and Treatment.* By John M. Johnson, M. D., of Atlanta, Ga.

The etiology of this disease is too obscure, and our knowledge of the remote origin of it too uncertain to warrant even speculation on the subject. It is true that the pathological conditions surrounding it, point unmistakably to nervous changes, and scarcely leaves room to doubt that it has its origin in nervous degeneration, beginning in the nerve cells of a particular centre, involving the filiments fed by them, and on the principle of election depositing the waste molecular matter in the skin, where the disease manifests itself, or what is equally probable, the functions of the minute nerves being impaired, inertia of the capilaries may follow, with congestion, and carbuncle as the result.

I have never seen carbuncle attack the thin skin. Generally it attacks the nape of the neck, back and nates. Out of one hundred and thirty cases treated by me, I have never seen one on any other locality. Instances of this are given, however. Boils and Erysipelas have the same pathological origin, and with modifications as to locality and violence, require the same general treatment.

Another theory, scarcely less plausible, is the presence of acids, both in the nerves and fleshy substance, as a consequence of mental or physical labor too long continued, or under too great difficulties. We might readily trace eruptive diseases, pneumonia, typhoid fever, &c., &c., ʇo this cause, but there would be difficulty in always locating carbuncle in the thick skin, and scarcely even in the thin parts of it, by this theory.

Certain it is; that preceding the developement of carbuncle, there is always considerable constitutional disturbance, as characterized by irregular pulse, flashes of heat, scanty and acrid urine, or it may be abundant and clear, variable appetite, headache, liability to colds, restlessness, unrefreshing sleep, nervous depression, &c., &c.

Carbuncle is inflammation of the true skin. It begins with a small red point, which rapidly swells to an enormous size. From the first it is pungently painful, and although a mere speck at the beginning, the pain embraces an extended surface, and is of the most aggravated kind and degree. The skin thickens to the extent of moıe than an inch. As it enlarges, the number of specks upon the surface increases. They discharge a yellowish matter in small quantities. These yellowish specks are found throughout the diseased skin, down to the healthy substance below, and is one of the prominent tests of its true carbuncular character.

*Treatment.*—After the development of carbuncle, if there has been no previous treatment, catharsis should be produced, if the bowels are sluggish, which is generally the case. After which :—

℞—Mild Chlo. Mercury, gr. x.
Sach. Alba., gr. x.
Make powders, x.

One to be given three times daily.

℞—Muriated Tinct. Iron, ʒ ii.
Quinine, ʒ i.
Mix and dissolve. Then add—
Aqua. pura. ℥ ii.
Mix. 1 teaspoonful every three hours.

In addition to this, a free crucial incision should be made through the thickened skin, and the gaping wound will make it apparent when you have reached the bottom; then place lint between the edges and a strong ley poultice over it, and healthy supuration, followed by healing, will take place.

For the purpose of perfect restoration I give for ten or fifteen days the following:—

℞—Strychnine, gr. ii.

Quinine, ʒ i.

Iron by Hydrogen, ʒ i.

Mix for pills, xxx.

One, three times daily.

I hold the belief that this family of diseases are of malarious origin, or owe their existence to like causes.

Light, uniritating, but nourishing food, should be given, with wines and cordials, if they do not disagree with the stomach or interfere with the comfort of the patient.

Prof. Syme objects to stimulants in this complaint. His objection is not well taken. Sometimes they are inadmissible; generally they are useful.

